# Effects of dietary plant-derived phytonutrients on the genome-wide profiles and coccidiosis resistance in the broiler chickens

**DOI:** 10.1186/1753-6561-5-S4-S34

**Published:** 2011-06-03

**Authors:** Hyun S Lillehoj, Duk K Kim, David M Bravo, Sung H Lee

**Affiliations:** 1 Animal Parasitic Diseases Laboratory, Animal and Natural Resources Institute, United States Department of Agriculture-Agricultural Research Service, Beltsville, MD 20705, USA; 2 Pancosma S.A., Voie-des-Traz 6, CH-1218 Le Grand Saconnex, Geneva, Switzerland

## Abstract

**Background:**

The present study was conducted to investigate the effects of dietary plant-derived phytonutrients, carvacrol, cinnamaldehyde and Capsicum oleoresin, on the translational regulation of genes associated with immunology, physiology and metabolism using high-throughput microarray analysis and *in vivo* disease challenge model of avian coccidiosis.

**Methods:**

In this study, we used nutrigenomics technology to investigate the molecular and genetic mechanisms of dietary modulation of host innate immunity and metabolism by three phytonutrients. To validate their immunomodulatory effects in a disease model, young broiler chickens fed a standard diet supplemented with three phytochemicals (carvacrol, cinnamaldehyde, and Capsicum oleoresin) from one day post-hatch were orally challenged with *E. acervulina*. The body weight gain and fecal oocyst production were used to evaluate coccidiosis disease parameters.

**Results:**

Analysis of global gene expression profiles of intestinal tissues from phytonutrient-fed birds indicated that Capsicum oleoresin induced the most gene changes compared to the control group where many of these genes were associated with those of metabolism and immunity. The most reliable network induced by dietary cinnamaldehyde treatment was related with the functions of antigen presentation, humoral immune response, and inflammatory disease. Furthermore, dietary supplementation with these phytonutrients significantly protected broiler chickens against live coccidiosis challenge infection based on body weight and parasite fecundity.

**Conclusions:**

The results of this study provide clear evidence to support the idea that plant-derived phytochemicals possess immune-enhancing properties in chickens and these new findings create a new possibility to develop effective drug-free alternative strategies for disease control for poultry infectious diseases.

## Background

In view of rising concerns on the extensive use of antibiotics in animal production, there is an increasing interest for developing alternative disease control strategy to enhance animal health and to reduce the use of antimicrobials. One promising new possibility to achieve this goal is the use of natural foods and herbal products to enhance feed efficiency, gut health, and innate immunity [1]. In clinical medicine, plant-derived products are increasingly being used as feed supplements to enhance immunity to diseases and cancers. Among these products, the dietary effects of the mixture of three plant-derived phytochemicals, carvacrol, cinnamaldehyde, and *Capsicum* oleoresin as anti-bacterial and anti-fungal agents have been reported [2]. Carvacrol is a component of numerous aromatic plants, such as *Origanum vulgare*, thyme, and wild bergamot [3]. The anti-microbial functions of these herbs are associated with carvacrol [4,5] since carvacrol vapour has been shown to inhibit *Salmonella* growth in chickens [6]. Cinnamaldehyde is a constituent of cinnamon and widely applied as flavoring. It has been proven to have strong anti-bacterial activity against *Escherichia coli*, *Pseudomonas aeruginosa*, *Enterococcus faecalis*, *Staphylococcus aureus*, *Staphylococcus epidermidis*, *methicillin-resistant Staphylococcus aureus* (MRSA), *Klebsiella pneumoniae*, *Salmonella sp.*, *and Vibrio parahemolyticus*[7]. Hot pepper (Capsicum spp.) is vegetable of importance in human nutrition and has many beneficial effects on human health [8,9]. *Capsicum* oleoresin, prepared by organic extraction of pepper fruits, contains anti-bacterial activity and is effective in treating stomach illnesses (Spices board, 2008). It contains the pungent principles, capsicin which has effects on the resistance to *Salmonella enteritidis* infection by altering pH and histological changes [10,11]. However, there is very limited information on the use of phytonutrients in veterinary medicine, and almost no knowledge on the underlying immunomodulation mechanism mediated by dietary phytonutrients in poultry.

With emerging “omics” technology, scientists are now better able to investigate how dietary food components can affect physiological functions and the underlying cellular and molecular mechanisms. Nutrition-related genomics technology has revolutionized the field of nutrition and two similar and yet distinct disciplines related to nutrition genomics have evolved, “nutrigenetics” and “nutrigenomics” [12]. In particular, the emerging field of functional nutritional genomics has provided unprecedented opportunities for increasing our understanding of how nutrients modulate gene and protein expression to influence cellular metabolism [12]. When integrated with other “omics” technologies in a systems biology approach, novel nutrition-based intervention strategies are expected to provide an effective alternative disease control strategy for agricultural animal industry.

In this study, we used three immunologically active phytochemicals (carvacrol, cinnamaldehyde and Capsicum oleoresin) to investigate the underlying molecular mechanisms of nutrition-mediated immunomodulation of host innate immunity and to validate their health promoting effects using an *in vivo* coccidiosis disease challenge model.

## Results

In the present study, we analyzed transcriptional profiles using the avian intestinal intraepithelial lymphocyte microarray (AVIELA) consisted of 10,162 spots. The total number of IEL elements which were significantly altered (> 2.0 fold) in the expression levels by three different phytonutrients (carvacrol, cinnamaldehyde, and Capsicum oleoresin) were 74, 62, and 254, respectively.

**Figure 1:**
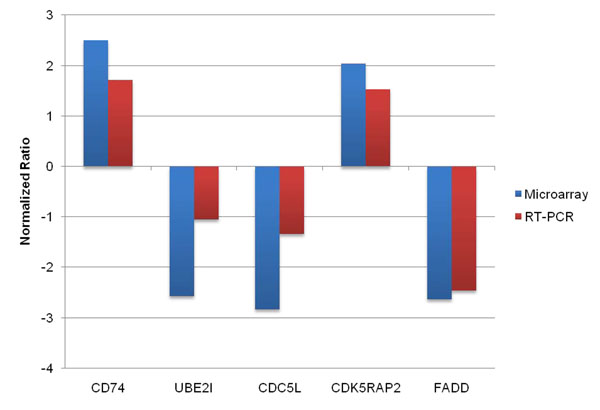
**Comparison between the expression levels of selected genes from microarray analysis and quantitative real-time PCR following dietary supplementation with *Capsicum* oleoresin**. CD74 molecule (CD74); ubiquitin-conjugating enzyme E2I (UBE2I); CDC5 cell division cycle 5-like (CDC5L); CDK5 regulatory subunit associated protein 2 (CDK5RAP2); Fas (TNFRSF6)-associated via de*a*th domain (FADD).

To confirm the results of microarray analysis, we selected five genes and followed the kinetics of their corresponding transcript levels following dietary supplementation with *Capsicum* oleoresin. All of the selected genes showed > 2.0-fold altered expression in the normalized AVIELA data (*P* < 0.05). Of these, two (CD74 and CDC5L) were associated with the first network and another two (UBE31 and FADD) were included in the second network of pathway analysis. As shown in Figure 1, the transcriptional changes in these genes as assessed by qRT-PCR showed similar patterns when compared with the original microarray data.

**Figure 2:**
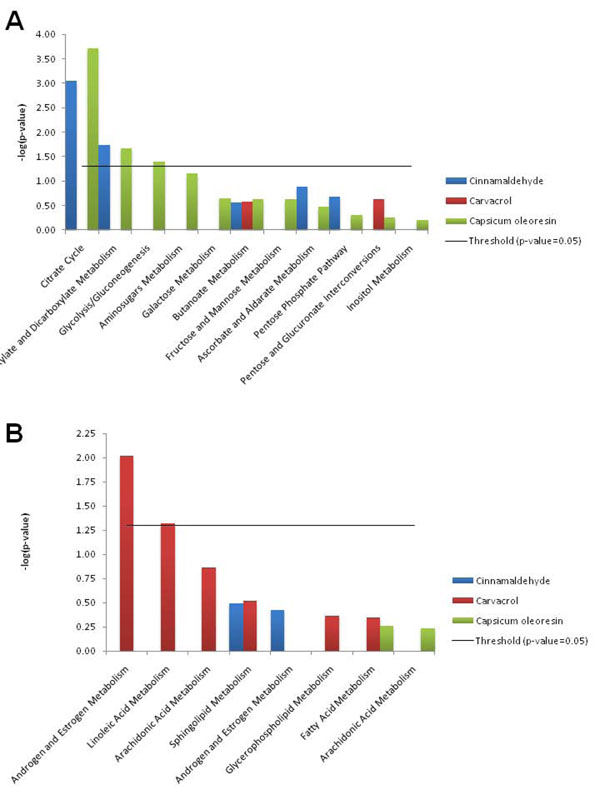
**Canonical Pathway analysis of differential expressed genes by the feeding of three different phytonutrients**. Datasets were analyzed by the Ingenuity Pathways Analysis software. The significance is expressed as a *P* value that is calculated using the right-tailed Fisher’s exact test. (A) The comparison of the pathways related with (A) carbohydrate metabolism and (B) lipid metabolism. Threshold: *P* value=0.05.

Pathway and gene network analysis using IPA software showed that Capsicum oleoresin and cinnamaldehyde significantly modified the pathways related with carbohydrate metabolism (Figure 2A) such as the citrate cycle (*P* values: 1.95 x 10^-4^, and 8.91 x 10^-4^, respectively), and glyoxylate and dicarboxylate metabolism (*P* values: 2.14 x 10^-2^, and 1.82 x 10^-2^, respectively). The pathway for glycolysis/gluconeogenesis was induced by Capsicum oleoresin (*P* value: 4.07 x 10^-2^). However, in lipid metabolism, only carvacrol treatment showed statistically significant changes associated with androgen and estrogen metabolism (*P* value: 9.55 x 10^-3^) and linoleic acid metabolism (*P* value: 4.79 x 10^-2^) pathways (Figure 2B).

**Figure 3:**
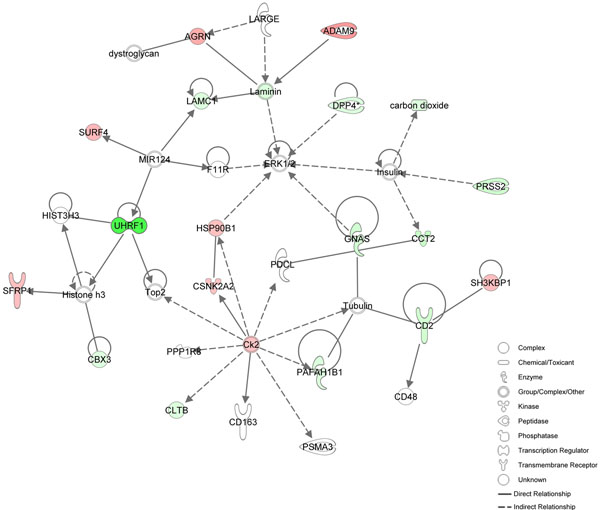
**The most reliable network of genes exhibiting >2.0-fold expression induced by cinnamaldehyde treatment**. Top functions of this network are Antigen presentation, Humoral immune response and Inflammatory disease. Up- and down-regulated genes are illustrated with red and green colors, respectively. The intensity of each gene indicates the expression level of the genes.

	IPA network analysis revealed that 7, 9, and 17 biologically relevant networks were associated with cinnamaldehyde, carvacrol, and Capsicum oleoresin groups, respectively. Among the networks, the three most reliable ones from each treatment were displayed in Table 1. Top functions represent the top 3 high-level functions from the Functional Analysis of a Network which represents an overview of the biological functions associated with a given network. Figure 3 describes the first network from the treatment group of cinnamaldehyde. This network includes 18 focus genes related to the functions of antigen presentation, humoral immune response, and inflammatory disease. All relationships between genes in the network are graphically represented as lines and nodes are displayed using various shapes representing the functional class of the gene product. These relationships are supported by at least one literature reference or from canonical information stored in the Ingenuity Knowledge Base.

**Table 1:** Three most reliable gene networks associated with each phytonutrient

Treatment	Network No.	Top Functions*	Focus Genes	Score
	1	Antigen Presentation, Humoral Immune Response, Inflammatory Disease	18	39
	
Cinnamaldehyde	2	Cardiovascular System Development and Function, Tissue Morphology, Drug Metabolism	14	28
	
	3	Cell Death, Gene Expression, Cellular Development	14	27

	1	Cell Morphology, Cellular Assembly and Organization, Cellular Function and Maintenance	14	29
	
Carvacrol	2	Cell-To-Cell Signaling and Interaction, Tissue Development, Cellular Movement	12	24
	
	3	Genetic Disorder, Renal and Urological Disease, Endocrine System Development and Function	12	24

	1	Gene Expression, Cardiovascular System Development and Function, Cellular Growth and Proliferation	23	39
	
Capsicum oleoresin	2	Developmental Disorder, Genetic Disorder, Neurological Disease	19	28
	
	3	Carbohydrate Metabolism, Cardiovascular System Development and Function, Hepatic System Disease	15	23

* The top 3 high-level functions from the Functional Analysis of a Network. This provides an overview of the biological functions associated with a given network.

**Figure 4:**
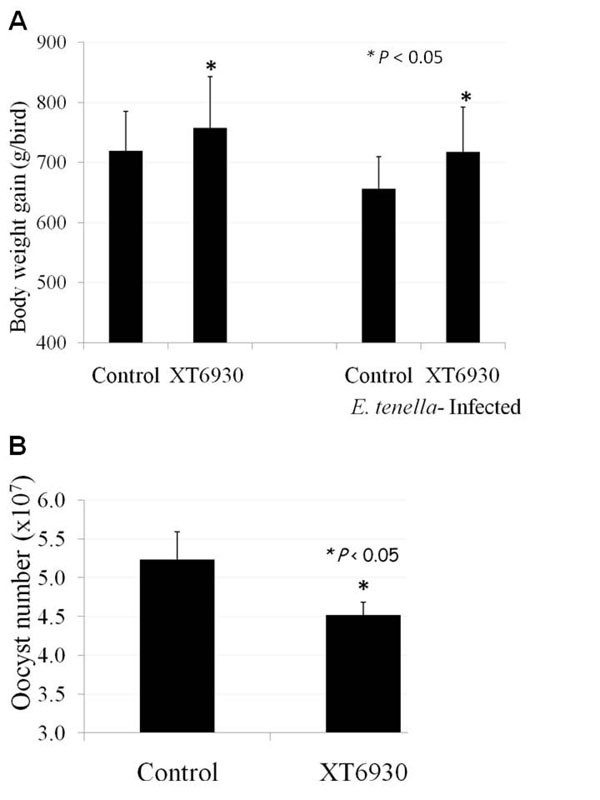
**Body weight gains (A) and total fecal oocyst output (B) of broiler birds fed with diet containing XT6930 (5 mg/kg of carvacrol, 3 mg/kg of cinnamaldehyde, 2 mg/kg of Capsicum) post *E. acervulina* infection.** Body weights were measured at 0 and 9 days post *E. acervulina* infection. Fecal oocyst outputs were collected during the 6-9 days post-infection period. *P<0.05

	To further validate biological functions of carvacrol, cinnamaldehyde, and Capsicum oleoresin in a poultry infectious disease model, broiler birds fed with standard diet supplemented with the plant extract mix designated as XT6930 (5 mg/kg of carvacrol, 3 mg/kg of cinnamaldehyde, and 2 mg/kg of Capsicum oleoresin) were orally challenged with *E. acervulina*. Feeding with XT6930 significantly enhanced body weight gains and reduced gut lesions of *E. acervulina*-infected chickens (Figure 4). Furthermore, broiler chickens which were continuously fed with a standard diet supplemented with carvacrol, cinnamaldehyde and Capsicum oleoresin from hatch showed significantly reduced gut lesions and lower pro-inflammatory cytokine gene expression (data not shown) post challenge infection with *E. acervulina* compared to the controls fed only the standard diet.

## Conclusions

This study demonstrates that dietary immunomodulation mediated by the combination of carvacrol, cinnamaldehyde and Capsicum oleoresin enhanced coccidiosis resistance. Microarray analysis indicated that these phytonutrients mediated transcriptional changes associated with metabolism and cell-mediated immunity which were reflected by enhanced protective immune response to avian coccidiosis. In general, Capsicum oleoresin induced most change of gene expression on AVIELA compared to the two other nutrients studied in this work.

Transcriptional profiling and pathway analysis revealed differential expression by the three dietary phytonutrients. The pathway analysis determined that lipid metabolism, linoleic acid metabolism and androgen and estrogen metabolism were regulated by carvacrol in this study. Carvacrol has well-known antibacterial and antifungal properties as well as antioxidant effects, with clinical applications for topical treatment of mucosal and epithelial infection [13,14]. In broiler meat, feed supplement with carvacrol was found to delay lipid oxidation after 5 and 10 days of storage [15]. Carvacrol inhibited the linoleic acid peroxidation as the same level as the positive control [16], influenced lipid metabolism and down-regulated the expression of oxidative stress markers such as CYP1A1 (cytochrome P450, family 1, subfamily A, polypeptide 1) [17].

In previous studies, supplementation with carvacrol, cinnamaldehyde, and *Capsicum* did not show any influence on body weights or feed efficiency, but improved ileal and fecal digestibility in homeostatic status in chickens [2,11,18]. However, in this study, dietary feeding of young broiler chickens with these nutrients improved body weight gain and reduced oocyst shedding in the diseased chickens. The detailed mechanisms are not known but may involve morphological modification of cells of the gastrointestinal mucosa [19] and genetic regulation of metabolic network. In our previous results [20], several immune-related genes showed changes following treatment with phytonutrients reflecting their well known medicinal effects against various bacteria or fungi in chicken infection studies [6,10,19]. Considering these observation, the phytonutrients used in this study have shown beneficial effects on host immune system and metabolic conditions through the regulation of gene expression in the chicken gut. Therefore, these nutrients have potentials to be the alternatives of antibiotics in poultry feed.

In the present study, the most reliable network of cinnamaldehyde treatment was determined as the functions of antigen presentation, humoral immune response, and inflammatory disease. Cinnamaldehyde has been known with its anti-inflammatory and anti-cancer effects [20,21]. In our previous study, the regulatory effects of cinnamaldehyde on inflammatory responses involved suppression of nitric oxide (NO) production, up-regulation of co-stimulatory molecules (CD80 and CD69) and proinflammatory cytokines such as TNF-α and IL-1β [22]. Among the focus gene including the first network altered by cinnamaldehyde treatment, CD2 which is a T and NK cell surface protein that mediates low affinity cell-cell interactions by binding to related immunoglobulin superfamily (IgSF) proteins, CD58 in humans and CD48 in rodents, was down-regulated [23]. Soluble DPP4 (dipeptidy l-peptidase 4) was reported to induce T cell proliferation in antigen presenting cells [24] and Hsp90b1 (heat shock protein 90, beta (Grp94), member 1) activates the release of nitric oxide by antigen presenting cells [25].

In conclusion, genome-wide profiling revealed differential expression by three dietary phytonutrients and bioinfomatical analysis identified the pathways and networks of genes induced by carvacrol, cinnamaldehyde and Capsicum oleoresin. These results provide new information concerning the molecular mechanisms involved in dietary modulation in the chicken gut and will facilitate the development of novel dietary strategies to enhance poultry health. Furthermore, for complex intestinal parasitic infection such as coccidiosis whose treatment has traditionally relied upon prophylactic medication, dietary immune enhancement using edible plants provides a safe alternative control method to reduce economic losses. With rapidly developing genomics technology, systems and network biology will play an important role to increase our understanding of how nutrition influences metabolic and immunity pathways and enhances animal health and well-being. Agricultural animal scientists should quickly adopt rapidly developing technologies to understand the underlying mechanism of nutrition-mediated immunomodulation and to develop novel dietary intervention strategies to achieve sustainable agriculture and to minimize the use of antibiotics in animal production. Furthermore, systems biology should be applied to investigate to what extent individual genetic variations affect animal’s response to nutrition and other environmental stress [26].

## Methods

### Experimental birds, diets and disease challenge

One-day-old broilers (Ross/Ross, Longenecker’s Hatchery, Elizabethtown, PA) were randomly assigned to 4 groups (20 birds per group). The proportions of carvacrol, cinnamaldehyde, and Capsicum oleoresin in feed were based on the optimal doses from our pretrial experiments [22]. Chickens were fed for 7 days beginning from hatch with a standard diet alone (control) or with diets supplemented with carvacrol (5.0 mg/kg), cinnamaldehyde (3.0 mg/kg), or Capsicum oleoresin (2.0 mg/kg). At 14 day post-hatch (dph), all birds except uninfected control were orally infected with 2.0 × 10^4^ sporulated oocysts of *Eimeria acervulina* (*E. acervulina*)*.* Body weights of chickens were measured at 0 and 10 days post-infection (dpi) as described [22].

### Quantitative RT-PCR (qRT-PCR)

Intestinal duodenum tissues were obtained from uninfected chickens (for microarray studies) at day 14 dph (4 birds/ group). To validate the microarray data, we selected 5 genes and determined the expression levels by qRT-PCR as previously described [27,28]. Total RNA was extracted using TRIzol (Invitrogen, Carlsbad, CA) and qRT-PCR oligonucleotide primers for chicken cytokines and the GAPDH internal control as previously described were used [27,28].

### RNA extraction and aminoallyl-labeled RNA preparation

Intestinal intraepithelial lymphocytes (IELs) were isolated by Percoll density gradient centrifugation as described previously [1]. Total RNA was isolated from pooled IELs from each treatment group using Trizol and aminoallyl-labeled RNA was prepared using the Amino Allyl Message Amp II aRNA Amplification Kit (Ambion, Austin, TX) according to the instruction of the manufacturer. Two 20-μg aliquots of each aminoallyl-RNA sample were fluorescently labeled with AlexaFluor 555 or AlexaFluor 647 (Invitrogen) and labeled RNA were column-purified using the RNA Amplification Kit (Ambion). The RNA concentrations and labeling efficiencies were determined spectrophotometrically.

### Mircroarray hybridization, scanning and image analysis

The avian IEL array (AVIELA) consisted of 10,162 spots representing elements from 3 sources: 1) cDNA from chicken IELs [29], 2) immune-related cDNA from the lipopolysaccharide-activated HD11 macrophage cell line [30], and 3) direct PCR clones of selected chicken cytokines and chemokines [29]. Each element was duplicated on the array slide. According to a reference design with dye swap [31], 4 values were obtained for each treatment. Hybridizations were performed using HybIt hybridization buffer (TeleChem, Sunnyvale, CA) in ArrayIt reaction cassettes at 50°C overnight as described [32]. Each sample had a repeated hybridization using the alternate fluorescent dye between the treatment and control. Images were acquired by laser confocal scanning using a ScanArray Lite microarray analysis system (Perkin-Elmer, Boston, MA) at a resolution of 10 μm. A 16-bit TIFF image was generated for each channel corresponding to the AlexaFluor 555 and AlexaFluor 647 dyes. The scanned microarray images for each channel were overlaid and fluorescent intensities were quantified using ScanArray Express 3.0 software (Perkin-Elmer). Spots were detected using an adaptive circle algorithm in the ScanArray program and all spots were visually confirmed.

### Microarray and bioinformatics data analysis

GeneSpring GX10 software (Silicon Genetics, Redwood, CA) was used to qualify and normalize image analysis data and to perform the fold-change analyses as described [32]. To generate signal ratios, signal channel values (treatment group) were divided by control channel values (control group). The significantly differentially expressed genes were filtered using the volcano plot built by comparing the treatment of each phytonutrient with itself at the false discovery rate (FDR) < 0.1. The modulated elements were defined by 2-fold differences and a cutoff of *P* < 0.05 by parametric test. The microarray information has been submitted online into the Minimum Information About a Microarray Experiment (http://www.mged.org/Workgroups/MIAME). The accession number for this study is E-MEXP-2204. IEL cDNA elements which were used to create the IEL cDNA microarray were mapped to the chicken genome reference assembly (version 2.1) and reference RNA and protein sequences (formatted database for Blast, December 2007) using National Center for Bioinformatics Institute (NCBI) Blast (version 2.2.13). To analyze pathway information, chicken Entrez gene identifications wer mapped to Homologen IDs (locus link IDs) for human genes because a large portion of these sequences have not been defined in chicken.

The genes which were differentially expressed by treatments were analyzed by the Ingenuity Pathway Analysis (IPA) software (Ingenuity Systems, Inc. Redwood City, CA). Each identifier was mapped to its corresponding gene object in the Ingenuity Knowledge Base. Both 2.0-fold up- and down-regulated identifiers were defined as value parameters for the analysis. These genes, called focus genes, were overlaid onto a global molecular network developed from information contained in the Ingenuity Knowledge Base. The functional analysis was performed to identify the biological functions, canonical pathways and the network of genes from the datasets that were mapped with the Ingenuity Pathways Knowledge Base. Fischer’s exact test was used to calculate a *P* value determining the probability that each biological function, pathway, and network assigned to that dataset was due to chance alone. Networks of focus genes were algorithmically generated based on their connectivity.

### Statistical analyses

Statistical analyses were performed using SPSS software (SPSS 15.0 K for Windows, Chicago, IL), and all data were expressed as means ± SEM values. Comparisons of the mean values were performed by one-way analysis of variance, followed by the multiple Duncan test and differences were considered statistically significant at *P* < 0.05.

## Authors' contributions

HSL conceived of the study, and participated in its design and drafted the manuscript. DKK carried out the microarray data analysis, the real time PCR, and helped to draft the manuscript. SHL performed the diets and disease challenge. DMB was involved in the experimental design and helped to draft the manuscript. All authors read and approved the final manuscript.

## Competing interests

The authors declare that they have no competing interests.
